# The risk and burden of smoking related heart disease mortality among young people in the United States

**DOI:** 10.1186/s12971-015-0041-z

**Published:** 2015-07-03

**Authors:** Rumana J. Khan, Christine P. Stewart, Sharon K. Davis, Danielle J. Harvey, Bruce N. Leistikow

**Affiliations:** Graduate group in Epidemiology, University of California, 5215 VM3A, One Shields Avenue, Davis, CA 95616 USA; National Human Genome Research Institute, Genomics of Metabolic, Cardiovascular and Inflammatory Disease Branch, Social Epidemiology Research Unit, 10 Center Drive, Bethesda, MD USA; Program in International and Community Nutrition, University of California, 3253B Meyer, One Shields Avenue, Davis, CA 95616 USA; Department of Public Health Sciences, University of California, One Shields Avenue, Med Sci 1-C, Davis, CA 95616-8638 USA

**Keywords:** Smoking, Younger adults, Mortality, Heart disease

## Abstract

**Purpose:**

Although cigarette smoking remains the most common risk factor for heart disease among the young, few studies have explored the relationship of smoking with heart disease mortality risk among young people. This prospective study assesses the risk and burden of all heart disease (HD) and coronary heart disease (CHD) mortality associated with smoking among younger adults from a nationally representative sample of the United States.

**Method:**

National Health Interview Survey respondents’ data from 1997–2004 were linked to their death records through 2006. The analyses were restricted to individuals 18 to 44 years of age during follow up (n = 121,284). Cox proportional hazard ratios (HR) were estimated with adjustment for sample weights and design effects. Attributable fractions (AF) of smoking were calculated.

**Results:**

After controlling for age, race, body mass index, history of hypertension and diabetes, and leisure time physical activity, current smoking related CHD mortality HR was 14.6 [95 % confidence interval or CI, 3.3–64.9] for females and 3.6 [95 % CI, 1.2–10.4] for males. The HR for all HD mortality was 3.1 [95 % CI, 1.3–7.6] for females and 2.4 [95 % CI, 1.2–4.7] for males. The AF of smoking for CHD deaths for female and male were 0.58 and 0.54 respectively. The AF of all HD mortality was 0.31 for male and 0.32 for female. The mean estimates of all HD deaths attributable to smoking during 1997–2006 among this age group were 52,214, of which 45,147 were CHD deaths.

**Conclusion:**

Even after adjustment for multiple risk factors and without addressing passive smoking, our result showed a strong relationship between smoking and HD and CHD mortality among young adults that is likely causal.

## Background

In the United States (U.S.), heart disease (HD) ranks the first among the leading causes of death [1]. This is also the most costly disease and the cost is increasing with time. The estimated health care spending and lost productivity from morbidity and mortality of heart diseases including coronary heart disease (CHD) rose from $258 billion in 2006 to $316 billion during 2010 [2]. It is projected that this cost may triple by the year 2030 [3]. The onset of HD particularly among younger adults can have particularly high costs. Because people in this age range are in the prime of their most productive years, the consequences of HD are clinically and financially significant, including premature death or disability, loss of employment, and reduced quality of life. The incident rate of HD may be lower at younger age, but HD diagnosed at early stage of life has an overall poor prognosis [4]. Moreover, young people are less likely to seek medical care and thus, asymptomatic cases of HD might be missed. This leads to a possibility that the prevalence of HD could be grossly underestimated among this group and the patients who seek medical attention due to symptomatic disease may represent the “tip of the iceberg”. Many investigators have identified current smoking as the single factor most strongly associated with all HD and specifically, CHD events among young individuals [5–7]. These studies reported that between 76 % and 90 % of young patients with CHD are smokers compared with 40 % of older patients [5–7]. Young patients are also characterized by lower proportion of hypertension, diabetes mellitus and lipid disorders, but higher proportion of heavy smoking [5].

Although cigarette smoking remains the most common risk factor for CHD among the young, few studies have explored the relationship of smoking with heart disease mortality risk among young people. Most studies done in this area are cross sectional or case control studies [8–11] with a few prospective ones [4, 12]. It is worth noting that most of these studies had non-fatal CHD as the outcome measure and thus mortality has not been definitively reported in a large series or from a representative sample. Mortality from all kind of HD is also absent from the current literature. We have tried to address these issues and the objective of this paper was to assess the risk and burden of all HD and CHD mortality associated with smoking among younger adults (18–44 years) from a nationally representative U.S. sample.

## Methods

Data for this analysis were pooled and combined from the 1997–2004 administrations of the National Health Interview Survey (NHIS). Details on the survey methods are available at the NHIS website [13, 14]. In short, the NHIS is an annual, cross-sectional household survey conducted by the Centers for Disease Control and Prevention, National Center for Health Statistics (NCHS) and is representative of the civilian non-institutionalized population of the U.S. [13, 14]. To achieve sampling efficiency, the NHIS survey uses complex, multistage probability sample design that incorporates stratification, clustering and oversampling of certain subgroups. The first stage of sampling involves dividing the U.S. into approximately 1,900 geographically defined areas called Primary Sampling Units (PSU). PSUs are grouped into strata using social and demographic characteristics of the area. One or more PSUs are sampled per stratum, with the probability of selection for each PSU being proportional to its population size within strata. In the next stage of sampling, a selection of geographic area segments is sampled from within each selected PSU. These segments are then subdivided into clusters, each of which contains a small number housing units. All households are then assigned a quarter of the year for interview and eligible members of these sampled households are invited to participate in the basic survey interview. We obtained information on participant’s demographic factors, race, body mass index or BMI (calculated as weight in kilograms divided by height in meters squared), smoking history (smoking behavior, age first smoked regularly and cigarettes per day), physical activity level, personal history of CHD risk factors like diabetes and hypertension from the “Sample Adult Core Questionnaire”, which was administered to one randomly selected adult (aged ≥18 years) from each family within a household. These data are publicly available at the NHIS websites [13–15]. We combined 8 years of NHIS (1997–2004) data and linked them with their death records available at National Death Index database, which provided mortality follow-up data from the date of the NHIS interview through December 31, 2006 [16–17].

To know about their smoking status, all the NHIS respondents were asked, “Have you smoked ≥100 cigarettes in your entire life?” and “Do you now smoke cigarettes every day, some days, or not at all?” Smokers were defined as those who had smoked ≥100 cigarettes during their lifetime. Current smokers were defined as smokers who reported smoking every day or some days. Past smokers were smokers who reported that they did not currently smoke. Those who didn’t smoke ≥100 cigarettes in their entire life were considered as never smokers. Thus, smokers were classified as never, current and past with never smokers being the reference. To get adequate cell counts for CHD deaths, smokers were also categorized as current and non-current smokers where, non-current smokers included never smokers plus past smokers. Mortality was categorized as all HD mortality and CHD mortality. NCHS cause of death coding for all U.S. deaths used the 10th revision of the International Statistical Classification of Diseases, Injuries, and Cause of Death (ICD-10) starting in 1999. Cause of death coding for all U.S. deaths occurring prior to 1999 follows the 9th revision (ICD-9) [17–19]. However, a new variable was created by NHIS as a recode of all deaths occurring prior to 1999 coded under ICD-9 guidelines into comparable ICD-10 based underlying cause of death groups. This variable was used to identify heart disease deaths. All HD mortality codes were I00 to I09, I11, I13, I20 to I51, and CHD mortality codes were I20 to I25 [17–19].

The analyses were restricted to individuals ≥18 years at the cohort entry and no more than 44 years of age at the end of follow up (participant’s death, turning 44 or December 2006, whichever came first). The analyses were further restricted to the participants with available smoking status and death code information. A total of 130,908 adults (aged 18–44 years) were interviewed during the NHIS survey years 1997 through 2004. Participants who were ineligible for mortality linkage (n = 8431), who didn’t have information on interview quarter (n = 559), who were unsure about their smoking status (n = 616) and who didn’t have any information on death codes (n = 18) were excluded from the analysis. The final data set included 121,284 participants, with 625,743 person-years (male 280,354 and female 345,389).

### Statistical Analysis

Analyses were done with adjustment for sample weights and design effects using the Stata statistical package to produce nationally representative results. The years being pooled (1997–2004) fell within the same sample design period with the same public use design variables, and according to the analytical guideline no changes were made to the design variables (Stratum and PSU) during this time period [18]. The pooled data were treated like one year of data with a very large sample size. The new eligibility adjusted sample weights provided on the NHIS Linked Mortality Files were used to prevent biased mortality estimates [19]. According to the recommendation of NCHS, sample weight adjustment of the pooled data set was done by dividing each sample weight in the pooled dataset by the number of years that are being pooled [19]. As eight (1997–2004) survey years of the NHIS data files were pooled, each sample weight was divided by eight for analysis purposes.

Descriptive statistics were used to summarize the characteristics of U.S. young adults in relation to their gender. For all other analyses, Cox proportional hazard analyses were performed using the Stata survival procedure. Person-time of follow-up accrued from the time interviewed through either the time of the death or the end of follow-up (December 31, 2006), whichever came first. Sex-specific and age-adjusted hazard ratio (HR) estimates and corresponding 95 % confidence intervals (CIs) of all HD and CHD disease deaths were reported for current and past smokers compared to never smokers. The same analyses were done to obtain the HR for the current smokers compared to non-current smokers (never plus past smokers). All multivariate models were adjusted for age in years, race (White, Black and others), BMI (continuous), history of hypertension (no/yes) and diabetes (no/yes), and leisure time physical activity (no physical activity/leisure-time physical activities for at least 10 min).

We calculated the smoking attributable fraction (SAF) of current smoking for all HD and for CHD by using the formula, [(HR_c_-1)*prevalence current smoking]/[1+ {(HR_c_-1)*prevalence current smoking], where HR_c_ = Hazard for current smoking [20]. The number of all HD and CHD deaths attributable to smoking in the U.S. population in the specified time period (1997–2004) was then estimated by multiplying the crude SAF by the total number of deaths of the specified age group (18–44 years) in the population during that time. Information on the prevalence of current smoking and the number of total deaths in age group 18–44 years for calculating SAF and deaths attributable to smoking were obtained of from the nationally published data sources [21–23].

## Results

Selected gender specific characteristics and smoking behaviour of the weighted study population of adults aged 18–44 years (n =121,284; representing an estimated 106 million young adults) in the NHIS from 1997 to 2004 are shown in Table 1. The mean age of the total population was 31.8 years at the interview and was similar for both males and females. The majority of U.S. young adults were white (79.8 %), which was slightly higher among males (81.0 %) than females (78.6 %). Prevalence of hypertension was slightly higher among males (10.4 %) than females (9.9 %). On the other hand, prevalence of diabetes was a little higher among females (2.0 %) than males (1.8 %). Females had somewhat lower BMI (mean, 25.8 and SE, 0.04) than males (mean, 26.8 and SE, 0.03) and they were also less physically active during leisure time than males (female, 64.9 % and male 69.9 %). The prevalence of current smoking was 28.9 % in men and 24.9 % in women. The mean age at smoking initiation was similar for both men and women (men, 17.1 years and women, 17.0 years). The mean number of cigarettes smoked per day were higher among males (15.5, 95 % CI, 15.3–15.8) compared to females (13.6, 95 % CI, 13.4–15.8).

**Table 1:** Characteristics of adults aged 18–44 years by gender in the United States: National Health Interview Survey, 1997-2004

Characteristics^a^	Male	Female	Total
	(N^A^ = 55,200)	(N^A^ = 66,084)	(N^A^ = 121,284)
	(N^w^ = 53,135,392)	(N^w^ = 53,069,053)	(N^w^ =106,204,445)
Age (In years)^b^	31.8 (31.7–31.9)	31.7 (31.6–31.8)	31.8 (31.7–31.9)
Race			
White	81.0	78.6	79.8
Black	11.8	14.2	13.0
Others	7.2	7.2	7.2
Known Hypertension	10.4	9.9	10.1
Known Diabetes	1.8	2.0	1.9
BMI^b,c^	26.8 (26.8–26.9)	25.8 (25.7–25.8)	26.3 (25.2–26.3)
Physically active during leisure time	69.9	64.9	67.4
Smoking status			
Never	57.5	62.1	59.8
Past	13.7	13.0	13.3
Current	28.9	24.9	26.9
Age first smoked regularly (years)^b,d^	17.1 (17.0–17.1)	17.0 (17–17.1)	17.0 (17.0–17.1)
Cigarettes per day^b^	15.5 (15.3–15.8)	13.6 (13.4–13.8)	14.6 (14.5–14.8)

Abbreviation: BMI, Body mass index

N^A^: Actual N or Actual sample in the data; N^w^: Weighted N or weighted/estimated sample after adjustment for sample weights and design effects

^a^Data represent percentage, except where noted

^b^Mean (95 % Confidence interval)

^c^Calculated as weight in kilograms divided by height in meters squared

^d^For past and current smokers

Results from the unadjusted and adjusted proportional hazards models for the risk of all HD and CHD mortality by smoking status (current and past smokers compared with reference group never smokers) are presented in Table 2. The multivariate model adjusted for age (years), race, history of hypertension, diabetes, BMI, and physical activity level showed that for all kinds of HD, female current smokers had a 4.4 [95 % CI, 1.7–11.7]) times greater risk of mortality compared to female never smokers and male current smokers had a 2.7 [95 % CI, 1.3–5.2]) fold increased risk compared to male never smokers. Female past smokers had a 3-fold increased risk of all HD mortality (HR, 3.4 [95 % CI, 0.9–12.9]) compared with female never smokers, but the p-value was only marginally significant (p = 0.07). No significant risk was found for male past smokers (HR, 1.6 [95 % CI, 0.5–5.4]). Male current smokers had a 5 fold increased risk of CHD mortality (HR, 5.3 [95 % CI, 2.0–14.7]) compared to male never smokers; the risk was also more than 3 times higher for male past smokers (HR, 3.2 [95 % CI, 0.6–17.6]), but it was not statistically significant (p = 0.17).

**Table 2:** Gender specific all heart disease and coronary heart disease mortality by smoking status^a^ among adults aged 18–44 years in the United States: National Health Interview Survey, 1997–2004 followed through December, 2006

Smoking Status^a^	Male (All HD)	Female (All HD)	Male (CHD)	Female (CHD)
Never Smoker (Ref)				
No. of deaths	21	12	7	3^c^
Current Smoker				
No. of deaths	34	22	22	13
Crude HR	3.2	4.8	7.7	
95 % CI	1.8–5.7	2.1–10.7	3.1–19.5	
Age adjusted HR	3.0	4.5	7.3	
95 % CI	1.7–5.5	2.1–10.0	2.9–18.3	
Mutivariate HR^b^	2.7	4.4	5.3	
95 % CI^b^	1.3–5.2	1.67–11.7	2.0–14.7	
Past Smoker				
No. of deaths	6	6	2^c^	3^c^
Crude HR	2.1	3.1	3.7	
95 % CI	0.7–6.1	0.9–10.3	0.7–20.1	
Age adjusted HR	1.7	2.5	2.8	
95 % CI	0.6–4.6	0.8–8.2	0.5–14.45	
Mutivariate HR^b^	1.6	3.4	3.2	
95 % CI	0.5–5.4	0.9–13.0	0.6–17.6	

Abbreviations: HD, Heart disease; CHD, Coronary heart disease; Ref, reference group; HR, hazard ratio; CI, confidence interval; No, number

Actual number of death from all causes for never, current and past smokers were 343. 367 and 102 respectively

^a^Smoking status categorized as never, current and past smokers

^b^Adjusted for age (years), race, history of hypertension, diabetes, body mass index, and physical activity level

^c^there were too few CHD deaths in females in categories of never smoker and past smoker to estimate HR and categories of never smoker and past smoker were collapsed in subsequent analysis

As there were a small number of CHD deaths in never and past smoker categories, they were combined and classified as non current smokers to provide more statistically valid and reliable HR estimates for current smokers (Table 3). Compared with the female non current smokers, the female current smokers had more than three times increased risk of dying from all HD (HR, 3.1 [95 % CI, 1.3–7.6]) and male current smoker had a 2.5 times increased risk of the same compared with male non current smokers (HR, 2.4 [95 % CI, 1.2–4.7]). The hazard of dying from CHD was much higher among female current smokers (HR, 14.6 [95 % CI, 3.3–64.9]) than female non current smokers. But our finding was somewhat limited by the fact that we had a small number of female deaths (6 deaths) in non current smokers, which was evident by our wide confidence interval.

**Table 3:** Gender specific all heart disease and coronary heart disease mortality by smoking status^a^ among adults aged 18–44 years in the United States: National Health Interview Survey, 1997–2004 followed through December, 2006

Smoking Status^a^	Male (All HD)	Female (All HD)	Male (CHD)	Female (CHD)
Non current Smoker (ref)				
No. of deaths	27	18	9	6
Current Smoker				
No. of deaths	34	22	22	13
Crude HR	2.7	3.6	5.3	10.4
95 % CI	1.5–4.7	1.8–7.3	2.1–13.2	2.9–24.2
Age adjusted HR	2.7	3.5	5.3	9.9
95 % CI	1.5–4.7	1.7–7.1	2.1–13.3	2.7–22.8
Mutivariate HR^b^	2.4	3.1	3.6	14.6
95 % CI	1.2–4.7	1.3–7.6	1.2–10.4	3.3–64.9

Abbreviations: HD, Heart disease; CHD, Coronary heart disease; Ref, reference group; HR, hazard ratio; CI, confidence interval; No, number

^a^Smoking status categorized as current and non-current smokers

^b^Adjusted for age (years), race, history of hypertension, diabetes, body mass index and physical activity level

Table 4 shows the crude and adjusted SAF of current smoking for males and females. The crude SAF for all HD deaths for current smoking were 0.31 for male and 0.32 for female, meaning that these were the estimated proportion of all HD death attributable to current smoking for males and females respectively. The SAF for CHD deaths were 0.54 for males and 0.58 for females. The mean estimate of all HD and CHD deaths in the U.S. attributable to smoking during 1997–2004 for age group 18–44 years were 52,214 (15,381 female and 36,833 male) and 45,147 (11,609 female and 33,538 male) respectively .

**Table 4:** Smoking Attributable Fraction (SAF) for current smoking and estimated numbers of deaths of all heart disease and coronary heart disease mortality among adults aged 18–44 years in the United States: National Health Interview Survey, 1997–2004 followed through December, 2006

	Male all HD	Female all HD	Male CHD	Female CHD
Crude SAF	0.31	0.32	0.54	0.58
Age adjusted SAF	0.31	0.32	0.54	0.56
Multivariate SAF^a^	0.27	0.28	0.41	0.71
Estimated deaths^b^	36,833	15,381	33,538	11,609

Abbreviations: HD, Heart disease; CHD, Coronary heart disease; SAF, Smoking Attributable Fraction

^a^Adjusted for age (years), race, history of hypertension, diabetes, body mass index, and physical activity level

^b^The number of all HD and CHD deaths attributable to smoking in the U.S. population was estimated by multiplying the crude SAF by the total number of deaths of the specified age group in the population during that time

## Discussion

We have documented here the risk and burden of HD mortality associated with smoking among adults aged 18–44 years from a nationally representative U.S. sample. After the adjustment for important confounding factors, the risk of dying from all HD for both, female current smokers and male current smokers were significantly higher than never smokers. This risk was almost similar when current smokers were compared to non current smokers. Like all HD, the risk of dying from CHD for female current smokers and male current smokers were also significantly higher than non current smokers. The prevalence of current smoking in age group 18–44 years during the years 1997–2004 was 26.87 % in our analysis. Though the prevalence showed a decrease in more recent years, it had always remained higher for younger age group than older group of people [21]. Smoking in young people was linked to the development of central adiposity and poor dietary patterns [24]. More importantly, it is believed that, specifically among the young, smoking can trigger CHD in individuals with minimal atherosclerosis or even with normal coronary arteries [25, 26]. All these factors together make young current smokers more vulnerable to cardiovascular diseases. Several studies also strongly suggest that amongst the conventional cardiovascular risk factors, current smoking is the most significant discriminating factor for HD among young adults [5–7].

In our study, the adjusted risk for all HD deaths were more than 2 fold and more than 4 fold higher in male and in female current smokers respectively compared to male and female never smokers. The association between current smoking and CHD compared to non-current smoking was even stronger. The hazard of smoking and HD mortality or HD events have been studied over the past several decades and the causative relationship between them is well established [8–12]. However, the effect of smoking on younger adult’s HD events or mortality has not been well documented. According to one prospective study, current smoking was one of the most important predictors of long-term mortality for CHD patients under age 40 regardless of treatment strategy [4]. The harm of current smoking is also higher in younger people than older age groups as different studies have reported an increased risk of developing CHD from smoking with decreasing age [11, 12].

Several studies have evaluated the impact of gender on the relationship between smoking and cardiovascular events or mortality. Some of them didn’t find any gender differentiation for young adults [9, 10]. However, all these studies were case control in design. On the other hand, several prospective studies emphasized differences by gender with a more harmful effect to women [12, 27, 28]. In one study, women below age 55 had almost double the risk for men of below 55 [12]. This difference almost leveled off for the older age groups [12]. A recent review of 26 studies found that, compared with nonsmokers, women who smoke have a 25 % greater risk of CHD than a male smokers, independent of other cardiovascular risk factors [29].

Though not statistically significant, all risk estimates in the current study were higher in women than in men and were not affected by adjustment for other major risk factors. Thus, our results also reinforced the previous finding that smoking conferred greater risk for women than men. It is important to note that, this increased risk among women cannot be explained by the difference in smoking practice or pattern between men and women because of evidence from our study and others. First, compared with men, the prevalence of current smoking in women was relatively low in our study. The literature also shows that, though the decline in smoking in young females has always not followed a linear trend, it was never higher than young men [30–32]. Second, the number of cigarettes smoked per day was also lower in women than men in our study and other national studies [32]. Finally, it could be argued that if substantially more men than women gave up smoking during follow up, that could explain some of the increased risk among women. We did not calculate information on quitting smoking. However, according to the estimates of NHIS 2005–2007, more women of age group 18–44 attempted to quit smoking in the past one year rather than men of the same age group. Thus, if the increased risk were due to smoking pattern of the individual, male smokers would have a greater risk of CHD than female smokers, which is contrary to the findings of our analysis and findings from other prospective studies [12, 27, 28]. These evidence lead to the conclusion that female current smokers had higher risk for CHD than male current smokers independent of other cardiovascular risk factors and individual’s smoking pattern, suggesting differences in mechanism of action of tobacco in men and women even at a young age. A possible cause of the sex difference could be an interaction of oestrogen hormone with components of the tobacco. Epidemiological evidence suggests that women who smoke are relatively deficient in oestrogen as constituents of tobacco smoke exert anti­oestrogenic effects [33, 34]. Thus the hormonal benefit of premenopausal women are reduced and oxidation of low-density lipoprotein (LDL) cholesterol in smokers is thus hampered [35]. This may increase the level of LDL cholesterol and decrease the level of high density lipoprotein (HDL) cholesterol [36]. Another possible explanation could be the presence of smaller coronary arteries than men [37]. As a result, smaller thrombus might be required for coronary occlusion in women, and thus the thrombogenic effects of smoking is magnified [38, 39]. These differences can also be a reflection of the overall gender disparity in receiving medical care for heart disease [40, 41].

We found both HD and CHD mortality to be strongly associated with current smoking only, and the magnitude of the risk is diminished with past smoking. This supports the benefits of quitting smoking. This is in agreement with findings from many previous studies that showed that smoking cessation reduced the risk of subsequent mortality and further cardiac events [42–45]. There has been debate as to the speed and magnitude of risk reduction of mortality when a smoker quits. Some studies found a meaningful reduction in risk after only 2 to 3 years [42, 44]. Some of the studies suggest that risk can decline to that of a lifelong nonsmoker [44, 45]. A systematic review of 23 prospective studies from different parts of the world strongly suggests that quitting smoking can reduce CHD mortality by 36 % [46]. They found this risk reduction relatively consistent, regardless of the factors like age or study population [46]. They also concluded that stopping smoking may have a greater effect on reducing the risk of mortality among patients with CHD who smoke than the effect of any other intervention or treatment [46].

A few limitations of our study need to be addressed. We assessed smoking status of individuals only at baseline. We were not able to get the updated smoking status of the participants over time. That is, some of our non-smokers might have had started to smoke as they were very young. It is also possible that some of our current smokers quit smoking and would have been classified as past smokers. Thus our study might have underestimated the risk as a recent study observed stronger associations of smoking with HD mortality when using updated information on smoking status [47]. We also were not able to calculate the effect of passive smoking on HD mortality. Individuals described as non-smokers could be at increased risk due to passive smoking, even if only occasionally exposed [48]. This also might lead us to an underestimation of the true risk. Another issue to be mentioned is, we had a small number of CHD deaths among never and past smoker categories and the categories were combined to provide more stable HR and SAF estimates. We acknowledge here the possibility of HR and SAF being underestimated by collapsing these groups and using them as reference. We were also not able to measure the dose response relationship and see the associations for light smokers versus heavy smokers due to limited number of deaths in this age group, especially for females. Finally, we did not have information on blood cholesterol, physical activity level and dietary habits of our participants and we could not control for them in our multivariable models. This might have confounded our estimates to some extent. However, we did control for other stronger cardiovascular risk factor like BMI, hypertension and diabetes mellitus and believe that our estimates were not affected by them.

## Conclusion

The results of this longitudinal analysis come from a representative sample and thus may be generalized to the U.S. population. We showed a strong relationship between smoking and HD mortality among young adults that is likely causal. The results also showed that smoking was more harmful to women than men with regard to HD deaths. As the precise mechanism for this risk difference between men and women is unclear, our results suggest the need for more exploratory study into the pathophysiology of HD, particularly CHD among younger women. Our results also emphasize that inclusion of a female perspective in smoking control policies is essential even among the young adults. It is also recommended that effective interventions should target young adults and should not only be directed towards preventing smoking initiation. People should also know the benefits of quitting and they should be encouraged to quit smoking at an early age.

## Abbreviations

U.S.: United States
HD: Heart disease
CHD: Coronary heart disease
NHIS: National Health Interview Survey
NCHS: National Center for Health Statistics
PSU: Primary Sampling Units
BMI: Body mass index
HR: Hazard ratios
CI: Confidence interval
